# Genetic evidence of broad spreading of *Lymantria dispar* in the West Siberian Plain

**DOI:** 10.1371/journal.pone.0220954

**Published:** 2019-08-20

**Authors:** Vyacheslav Martemyanov, Roman Bykov, Marya Demenkova, Yuri Gninenko, Sergei Romancev, Ivan Bolonin, Ilia Mazunin, Irina Belousova, Yuri Akhanaev, Sergey Pavlushin, Polina Krasnoperova, Yury Ilinsky

**Affiliations:** 1 Institute of Systematics and Ecology of Animals SB RAS, Novosibirsk, Russia; 2 Biological Institute, National Research Tomsk State University, Tomsk, Russia; 3 Institute of Cytology and Genetics SB RAS, Novosibirsk, Russia; 4 All-Russian Research Institute for Silviculture and Mechanization of Forestry, Pushkino, Moscow Region, Russia; 5 FSI "Russian Centre of Forest Health", Novosibirsk, Russia; 6 Baltic Federal University, Kaliningrad, Russia; 7 Center for Life Sciences, Skolkovo Institute of Science and Technology, Skolkovo, Russia; 8 Siberian Federal University, Krasnoyarsk, Russia; 9 All-Russian Plant Quarantine Center, Bykovo, Moscow Oblast, Russia; Texas State University, UNITED STATES

## Abstract

Gypsy moth *Lymantria dispar* L. 1758 (Lepidoptera: Erebidae) is one of the most dangerous forest pests of the Holarctic region. Outbreaks of gypsy moth populations lead to significant defoliation of local forests. Within the vast territory of the West Siberian Plain, we noted the outbreak front movement in the north-east direction with a speed 100–200 km per year. The reason for the outbreak’s movement is still unclear because *L*. *dispar* females are characterised by limited flight ability, which is not enough to support that movement *per se*. Herein, we analysed the mtDNA divergence pattern among *L*. *dispar* populations collected from the vast territory of the West Siberian Plain to determine the boundaries of populations and reveal the effect of the outbreak’s front movement on mtDNA patterns of populations. The 590-bp region of the cytochrome oxidase subunit I gene of the mitochondrial genome was sequenced for 220 specimens that were collected from 18 localities along a transect line (~ 1400 km). Our results clearly show that the gypsy moth populations of the vast Siberian territory are not subdivided. This result can be explained by extensive genetic exchange among local populations. Taking into account that the flight ability of *L*. *dispar* females is rather limited, we suggest that spreading occurs through ballooning of early instar larvae. This hypothesis was confirmed by the coincidence of the outbreaks’ movement direction with that of the dominant winds, complemented by the observation of ballooned larvae far from a forest edge.

## Introduction

The spatio-temporal distribution of animal populations is an important topic for population ecology especially when populations of animals are able to significantly fluctuate. Many insect species are often considered as the model taxonomic group for population ecology studies. They are one of the most numerous animals on the Earth and widely distributed, while also being the main consumers of green biomass in the biosphere [1]. Many herbivorous insects can produce population outbreaks. Cyclic population dynamics and their genetic consequences have been an area of interest in ecology for many years [2].

Gypsy moth *Lymantria dispar* L. 1758 (Lepidoptera: Erebidae) is one of the most dangerous forest pests of the Holarctic region, infesting over 300 plant species [3, 4, 5]. Outbreaking gypsy moth populations occupy vast territories that lead to significant defoliation of local forests [4, 6, 7]. An important aspect of *L*. *dispar* population dynamics is the regularity of outbreak emergence. The population dynamics of *L*. *dispar* is generally cyclical with a period of seven to nine years and is characterised by high amplitudes of population fluctuations. Some long-term shifts in the outbreaks cyclicity were found [8] based on the analysis of long-time series data (≥ 10 cycles) analysis. Usually, outbreaks of forest defoliators emerge synchronously at large spatial scales [9]. The travelling waves of outbreaking populations over geographical space is an alternative phenomenon that has been demonstrated by certain forest defoliators [10].

For European and North American *L*. *dispar* populations, the synchronous character of outbreaks has been shown [11, 12, 13, 14]. However, our observation of West Siberian (Asia) populations of *L*. *dispar* shows an asynchronous character of outbreaks. In addition, we noted for some regions the cases of travelling waves with the north-east direction (see the Results). South of West Siberia is a vast area of *L*. *dispar* range in the forest-steppe zone characterised by homogenous environmental conditions. In contrast to European *L*. *dispar* population in which females cannot flight, females of West Siberian *L*. *dispar* moth possess a flight ability [15].

The spreading adults *L*. *dispar* females cannot solely explain outbreak movement in West Siberia plain. The maximum of *L*. *dispar* female despersal does not exceed 10 km/season [16, 17, 18, 19, 20]. On the other hand, we observed the rate of pest outbreak movement in West Siberia plain was 100–200 km/season, which means there are additional reasons for outbreak movements.

In the present work, we aimed to explain the outbreak movement phenomenon by the analysis of mtDNA divergence patterns of populations collected from the vast territory of the West Siberian Plain in 2015–2016. In particular, we investigated the mtDNA divergence patterns of *L*. *dispar* populations: *i*) to determine boundaries of populations of Western Siberia, and *ii*) to compare mtDNA patterns between populations being at different phases of population cycle. There has been extensive collection of mtDNA data from *L*. *dispar* populations [21, 22, 23, 24, 25]. This allows us to compare our dataset with the genetic variation in mtDNA data of European population where there are no reports of outbreaks movement phenomenon.

## Methods

### Insect collection

**Fig 1:**
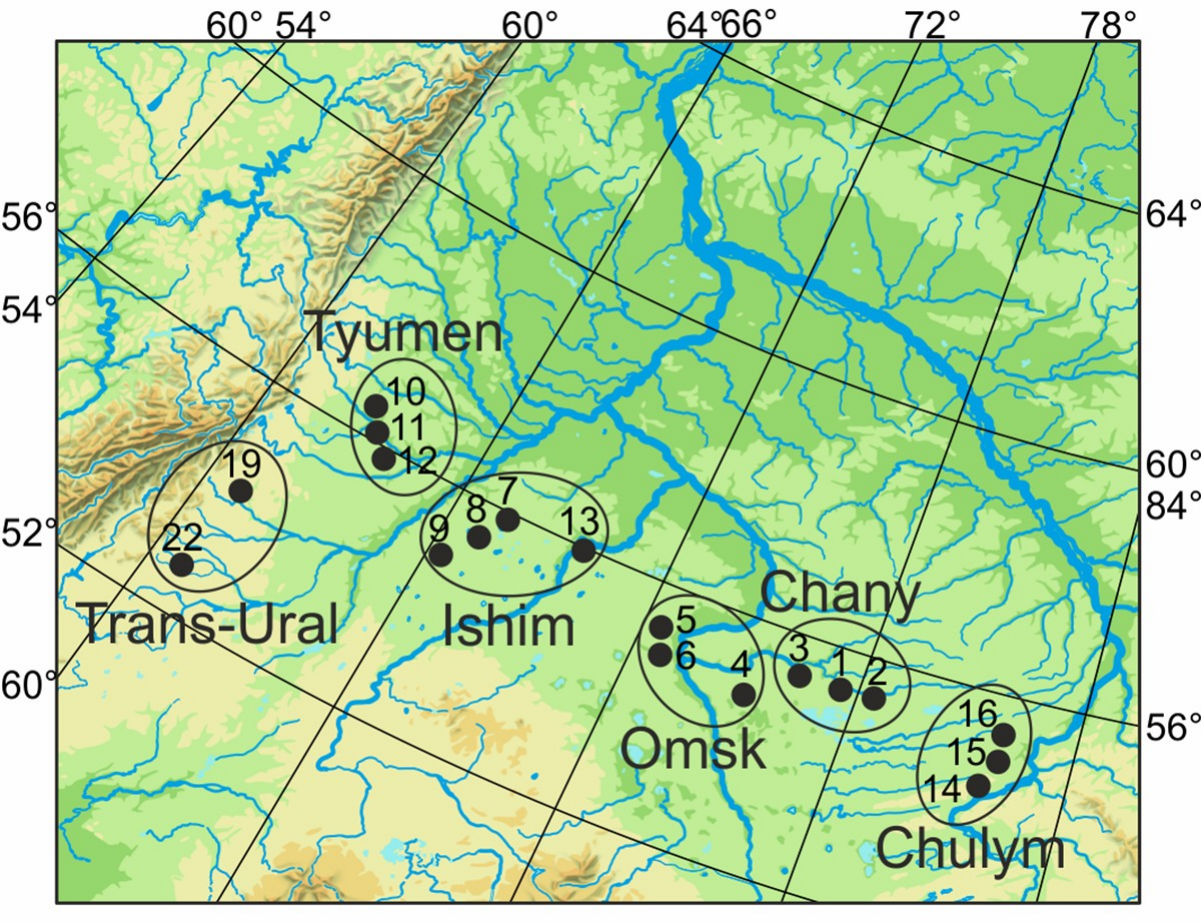
Map of *Lymantria dispar* collection in 2015–2016 seasons.

Insects were collected at the pupae or adult stages in July 2015–2016 in the West Siberian plain (Fig 1, Table 1). The sampling transect line was approximately 1400 km and included 18 localities.

**Table 1:** Localities, population phases and mtDNA haplotypes in collection of West Siberia plain territory of 2015–2016 seasons.

Population, locality name*	Coordinates	Phase of population cycle, year	N	Number of haplotypes
**Chulym**				
Shaidurovo (14)	N54.29 E81.15	Rising, 2015; 2016	12	I 5, II 5, III 2
Bazovo (15)	N54.34 E81.13	Rising, 2015; 2016	26	I 14, II 8, III 3, IIe 1
noname (16)	N54.55 E80.52	Rising, 2015; 2016	12	I 5, II 2, III 3, IIIb 1, IIIc 1
**Chany**				
Starye Karachi (1)	N55.28 E77.02	Rising, 2015	4	I 2, II 1, IIIa 1
noname (2)	N55.30 E77.10	Rising, 2015	1	I 1
Chany (3)	N55.24 E76.49	Rising, 2015; 2016	16	I 5, II 4, III 6, IV 1
**Omsk**				
Tatarsk (4)	N55.10 E75.53	Peak, 2015; Decline 2016	23	I 9, II 10, III 2, Ia 1, IIa 1
Krasny Yar (5)	N55.13 E72.53	Decline, 2015; 2016	20	I 9, II 7, III 2, Ib 1, IIb 1
Lubinsky (6)	N55.10 E72.43	Peak, 2015; Decline, 2016	15	I 4, II 7, III 3, IIa 1
**Ishim**				
Novolokti (7)	N56.03 E69.13	Decline, 2015	2	I 1, II 1
Loktyash (8)	N55.56 E68.54	Decline, 2015	1	II 1
Berduzhie (9)	N55.48 E68.23	Decline, 2015; 2016	10	I 6, II 3, IIIa 1
Bolshoy Krasnoyar (13)	N56.30 E67.45	Decline 2015	2	I 1, II 1
**Tyumen**				
Kyshtyrla (10)	N56.57 E65.44	Decline, 2015	1	I 1
noname (11)	N56.49 E65.50	Decline, 2015; Peak, 2016	30	I 14, II 12, III 2, IIc 1, IId 1
Kirovskiy (12)	N56.42 E65.42	Decline, 2015; Peak 2016	33	I 18, II 8, III 6, Ic 1
**Trans-Ural**				
Kamensk-Uralsky (19)	N56.47 E61.73	Troughs, 2015	5	I 2, II 2, III 1
Chebarkul (22)	N54.75 E60.30	Troughs, 2016	7	I 1, II 2, III 3, IIf 1

Footnotes:

* Number in brackets is an internal sample site.

We characterised each locality in terms of population cycle phase, and based on it, the transect line was subdivided into six areas, referred to as ‘populations’. Distance between nearby populations was 150–200 km. Following criteria were used for the characterisation of *L*. *dispar* population phases. *i*) The ratio of previous/current year egg masses. It is easy to do in West Siberia in comparison to other regions because here females lay egg masses on the base of the tree stem that will be covered by a snow layer); *ii*) severe defoliation which shows the peak phase. *iii*) the heavy weight of female pupae means the rising phase because heavy females of *L*. *dispar* have higher fecundity (features of aphages adults) while small size *vice versa* the decline phase. *iv*) The high amount of parasitized larvae/pupae (cocoons of parasitic wasps around larvae or pupae with typical holes from flies) indicating the decline phase. The rising phase was characterized by the bigger number of new egg masses then old ones; females pupae were heavy (heavier than 1.5 g) and parasitism level was low. The peak was characterized by severe defoliation of birch stands with extremely high numbers of egg masses (10-40/tree). The decline phase was characterized by the fewer number of new egg masses than old ones; the size of female pupae/exuvium was small (less than 1 g or equal sizes for exuvium) and parasite abundance was high. The “troughs” phase was assigned when extremely low population density (< 0.005 egg mass/tree) was registered.

In the outbreaking populations, insects were collected by net (males) or by hand (females). For low population densities (i.e., trough phase), adult gypsy moths were caught via pheromone or light lures. Adults were stored in 95% ethanol. No permits for a field collection are required for this study, since the national forests in Russia are freely accessible. No protected species have been sampled.

### Spatial-temporal distribution of *L*. *dispar* outbreaks

**Fig 2:**
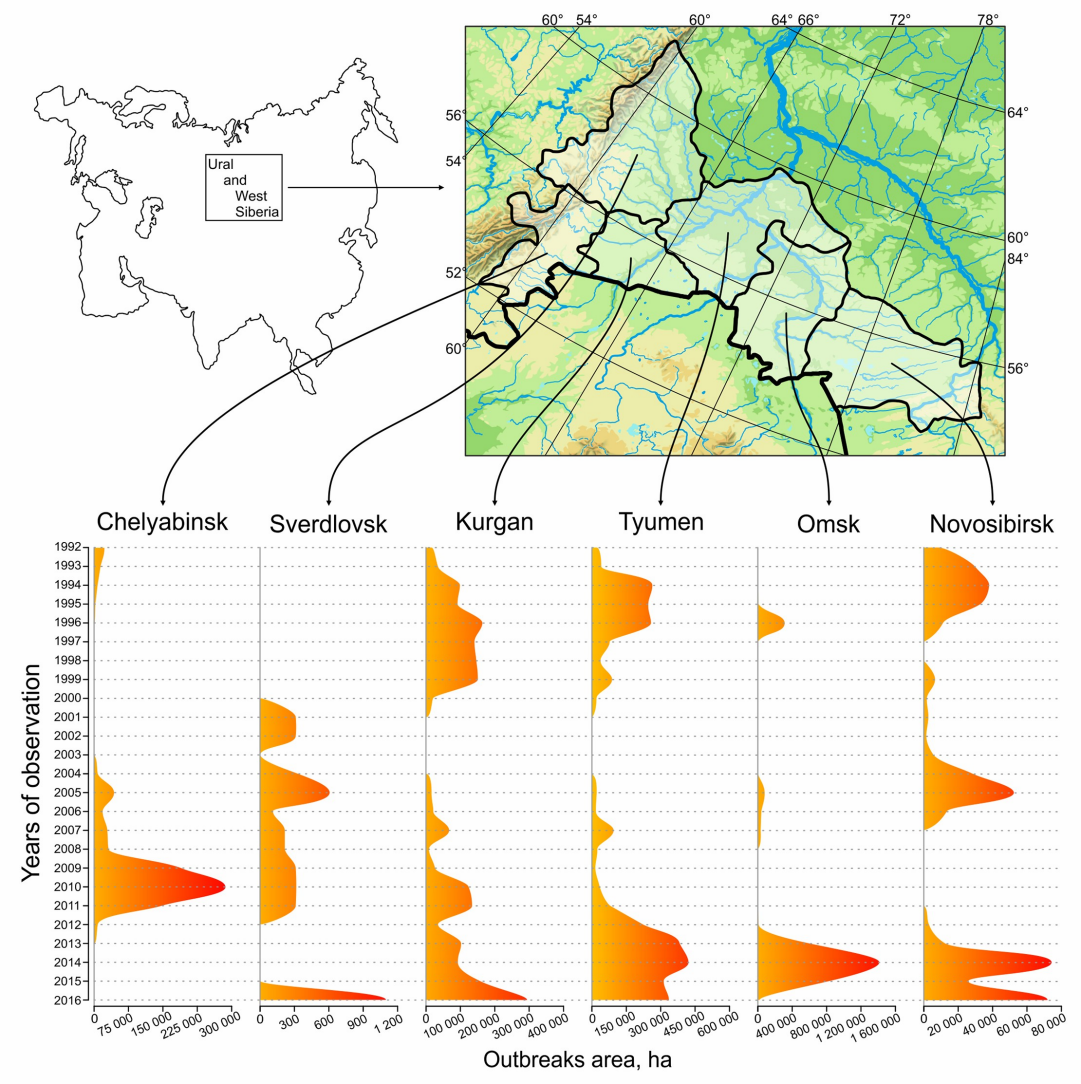
Time-series data of *Lymantria dispar* outbreak areas in administrative oblasts of Russia in West Siberia.

To determine the spatial-temporal distributions of pest outbreaks, we considered time-series data of gypsy moth densities, measured as the hectares of defoliated forest per year in the Sverdlovsk, Chelyabinsk, Tyumen, Kurgan, Omsk, and Novosibirsk oblasts (Fig 2). Initial data and measurements of defoliation level in accordance with [26] were officially presented by the Federal Agency for Forestry of Government of Russia. For calculation, we included the areas of forests where average defoliation levels exceeded 25% [26]. We utilised spectral analysis to calculate the cyclicity of outbreaks, a basic method for assessing cyclic oscillations across a time series [27, 28, 29]. The presence of a peak in the spectral density indicates the existence of cyclic components in a time series In order to calculate the spectral density function of our time series we transform the original time series to stationary. In this case its mean values and standard deviation do not change over time.

To reduce the dispersion of the studied time series and switch over to the logarithmic scale, all values of the defoliation areas, *x (i)*, were replaced by *x´ = x + 1*, which permitted us to transform the data with zero values of defoliation areas correctly: if *x* = 0, then *ln x = -∞* but *ln x´ = ln (0 + 1) = 0*.

Further, with the analysed time series, it was necessary to remove high-frequency noise. The Hunn filter was applied for this purpose [30]:

L(x(i))=0.24x(i‐1)+0.52x(i)+0.24x(i+1).

Removing high-frequency noise allows filtering high-frequency components of the time series with frequencies higher than 0.25/ year. The carried out transformation of the time series allows to work correctly with the data in the program, Statistica 10.0.

To assess the coherence of time series in population dynamics, the cross-correlation function is used. The cross-correlation function, *p*_*xy*_ *(k)*, can be calculated for two stationary time series, x and y, with mean values, *μ*_*x*_ and *μ*_*y*_, and standard deviations, *σ*_*x*_ and *σ*_*y*_ [27]:

$$P_{xy}\left(k\right)=\left(E|\left(x\left(t‐k\right)‐\mu_{x}\right)*\left(y\left(t\right)‐\mu_{y}\right)|\right)/\sigma_{x}\sigma_{y}$$

where E is the operator of the mathematical expectation and *k = 0*, *k* = *± 1*, *k* = *± 2* is the time delay (lag).

The absence of a time delay indicates that the time series are synchronous such that *k* = 0 and the value *p*_*xy*_ *(0)* → 1. The time series are coherent with a delay equal to the value of *k* if the maximum of the cross-correlation function falls on the value, *k* = *± 1*, *k* = *± 2*, etc. and the value of the cross-correlation function, *p*_*xy*_ *(k)* → 0, for any values of *k* for non-conjugate time series^27^.

For a more visible demonstration of outbreaks moving, we used the detailed data of the vicinities of the Novosibirsk oblast for generation of a movie (see S1 Video and S1 Fig). The outbreaks area data were provided by the Forest Agency of Russia and the Novosibirsk branch of the Forest Protection Service.

### DNA extraction, amplification, and sequencing

Total DNA was extracted from a leg of each specimen by incubation of homogenate in digestion buffer (see [31]). The 590-bp region of the cytochrome oxidase subunit I (*COI*) gene was sequenced for 220 specimens. The first part of the mtDNA amplicons (46 samples) was produced with a primer set, LepF1/LepR1, according to the original protocol [32]. A second part (174 samples) was created with the primer set specific to the *L*. *dispar* mitochondrion genome, LepF2 5′-TACCGCTTAAACTCAGCCAT-3′ and LepR2 5′-GAGGTAAAGTAAGCTCGTGT-3′, which allowed a more effective acquisition of amplicons. The LepF1/R1 primer set produced an amplicon of the 1511–2168 mtDNA region according to GenBank Accession No. FJ617240 and LepF2/R2 produced a 1457–2363 region. PCRs were carried out in a 30-μL volume with ‘BioMaster HS-Taq PCR (2x)’ (BioLabMix, Novosibirsk, Russia) with PCR cycling at 95°C for 5 min, 35 cycles at 95°C for 15 s, 55°C for 30 s, 72°C for 1 min, and final elongation at 72°C for 5 min. The amplicons were purified via the Zymoclean Gel DNA Recovery Kit (Zymo Research, USA) according to the manufacturer’s instructions and sequenced with an automatic capillary sequencer with PCR primers under the BigDye v. 3.1 (Applied Biosystems) protocol.

Nucleotide sequences of the *COI* gene were deposited in GenBank under accession numbers MK041668—MK041887.

### Analysis of mtDNA data

To determine the boundaries of L. dispar populations of West Siberia plain we used a mitochondrial marker, in particular, a part of cytochrome oxidase I subunit (COI) gene. A mitochondrial marker indicates maternal inheritance of a studied population, therefore migration activity of adult males between populations in the previous season could be neglected. Moreover, males of current year collection were collected in localities in a period when they emerged, so taking into account the design of the study adult males could not migrate in considered season. The main evolution factor that affects the genetic pattern in our case should be a genetic drift. Indeed, since the local populations regularly suffer dramatic changes in effective population size, the genetic drift should strongly influence mtDNA variation Therefore we assumed that mtDNA variation would be different in different populations. As to other evolution factors, mutations have extremely low rate and close or identical mitochondrial haplotypes are found in distantly related populations of modern L. dispar range; and positive selection of mtDNA has extremely low probability [33].

The alignments of nucleotide sequences were generated by the MUSCLE [34] that was integrated into Mega6 software [35]. DNA polymorphism: number of polymorphic sites (S), number of haplotypes (h), haplotype diversity (Hd), nucleotide diversity (Pi); and population analysis: values of Tajima D, Fu’s Fs, and Fst were performed using DnaSP v5 [36]. A TCS gene network [37] was performed by PopArt [38] to represent genealogical relationships among haplotypes and those frequencies.

To consider our results in contexts of populations that had no experience of so dramatic changes of population size we collected data of European *L*. *dispar* populations. Populations of Europe and West Siberia are distant and genetic exchange is unlikely. The taken European area was characterised by an uninterrupted range of *L*. *dispar* and by similar scale. Populations of European territory were quite well characterised in a relation of mtDNA variation of *L*. *dispar* (S2 Fig), and there was no effect of strong inbreeding on the population to compare with North American populations of *L*. *dispar* [23]. The dataset of Europe populations was retrieved from BOLD Systems (www.boldsystems.org) (see accession numbers in S1 Text).

## Results

### Spatial-temporal distribution of *L*. *dispar* outbreaks

**Fig 3:**
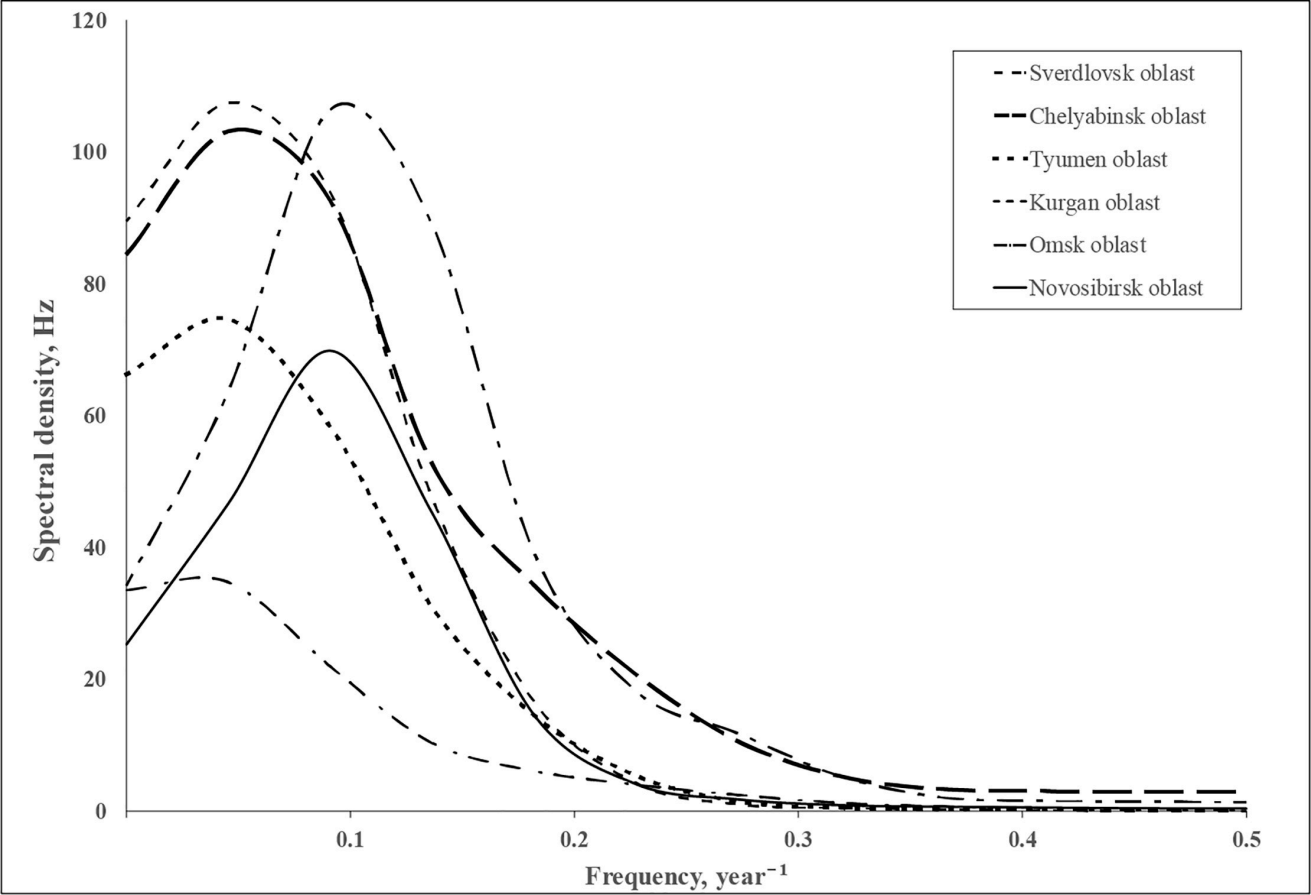
Spectral densities of log-transformed time series of squares of defoliated areas (ha).

Time series data of *L*. *dispar* outbreaks collected in the West Siberia territory (~1400 km) for 25 years have demonstrated that there is a cyclic component in a temporal context (Fig 3). In particular, the peaks of the spectral power for Sverdlovsk, Chelyabinsk, Tyumen, and Kurgan regions were the same and the frequency was f_max_ = 0.045/year, meaning that the between-peak period (L = 1/f_max_) was 22 years. For the Novosibirsk and Omsk oblasts, cycles were twice as frequent (Table 2).

**Table 2:** The characteristics of spectral density for the time series of square of defoliated forests after *L*. *dispar* outbreaks occurred in West Siberia.

oblasts	Frequency f_max_ of maximum of spectral density	Value of peak of spectral density maximum	Cyclicity of outbreaks L = 1/f_max_. years
Sverdlovsk	0.045/year	34.7	22.2
Chelyabinsk	0.045/year	103.1	22.2
Tyumen	0.045/year	74.6	22.2
Kurgan	0.045/year	107.5	22.2
Omsk	0.09/year	106.5	11.1
Novosibirsk	0.09/year	69.8	11.1

The numbers show the following names of administrative regions: 1—Sverdlovsk oblast, 2—Chelyabinsk oblast, 3 –Tyumen oblast, 4 –Kurgan oblast, 5 –Omsk oblast, 6 –Novosibirsk oblast.

**Fig 4:**
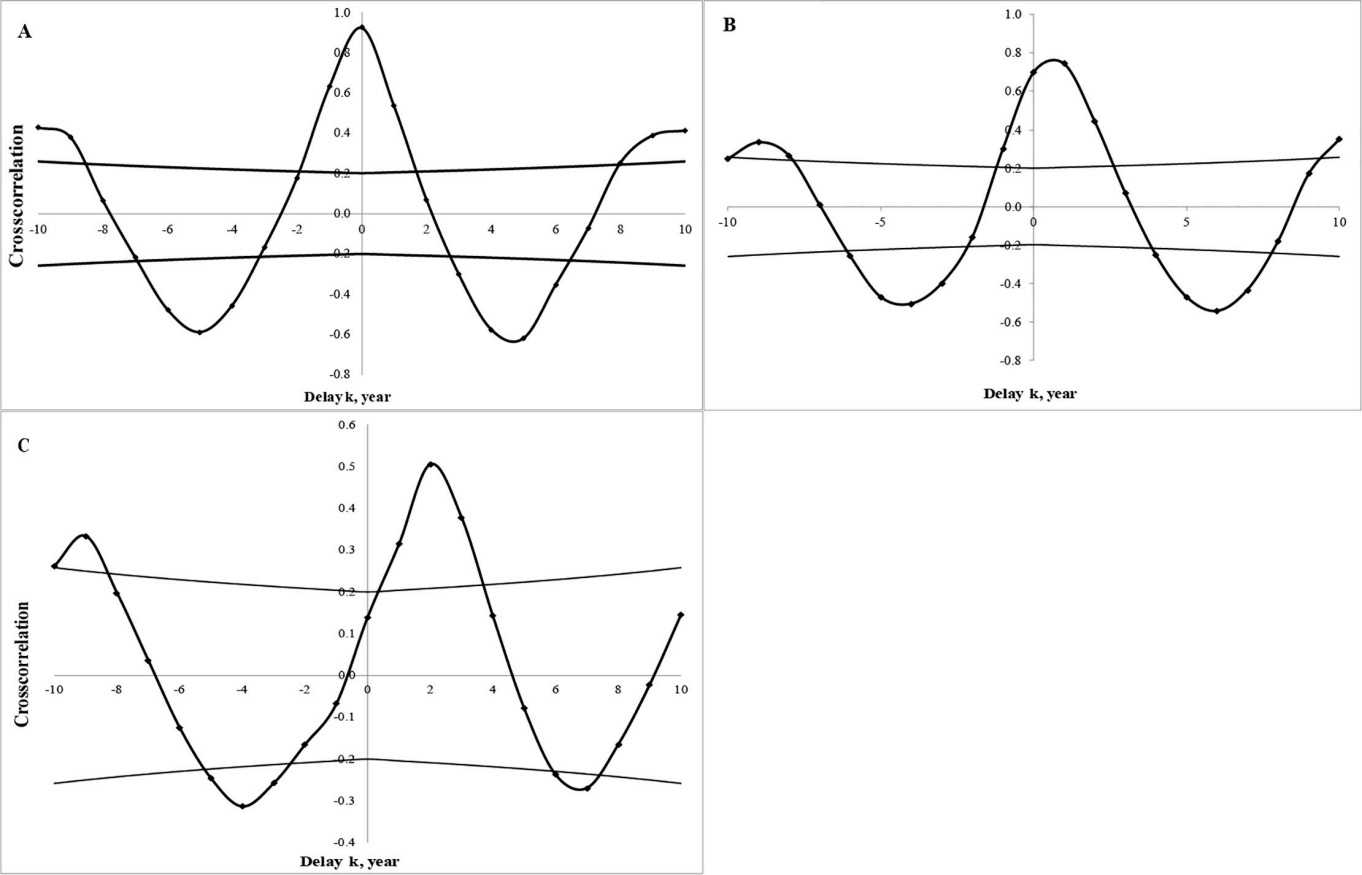
The cross-correlation function between time series data of forest defoliation (ha) for Novosibirsk oblast. The shift of function indicates the temporal delay in the north-east direction: for Karasuk and Kupino districts (a), Karasuk and Krasnozerskiy districts (b), and Karasuk and Kujbyshev districts (c).

No strict synchrony for outbreaks in the studied areas was observed (Fig 2). The statistical analysis of the cross-correlation function shows that outbreaks were mostly coherent between different regions, and this indicated the temporal delay between comparing areas (Table 3). It was difficult to ascertain the particular direction of spatial-temporal distribution of outbreaks for a large scale area. Whereas it was easier to determine when outbreaks were analysed over a smaller area. For example, the temporal delay of outbreak movement in the north-east direction was illustrated for the Novosibirsk oblast (S1 Table, S1 Video, S1 Fig, Fig 4). Thus, we provided evidence for the travelling wave phenomenon for outbreaking populations of *L*. *dispar*, which concurred with the direction of dominant winds during the spring period in Novosibirsk oblast.

**Table 3:** The characteristics of cross-correlation functions between defoliated areas of studied oblasts of the Russian Federation; the values of function/t-value are above the main diagonal and the temporal delays (k, years) are below.

	Sverdlovsk	Chelyabinsk	Tyumen	Kurgan	Omsk	Novosibirsk
Sverdlovsk		0.69/3.17*	0.60/2.5*	0.59/2.42*	0.21/0.98	0.66/2.95*
Chelyabinsk	-2		0.66/2.95*	0.65/2.94*	0.35/1.32*	0.56/2.17*
Tyumen	-6	-3		0.97/4.65*	0.51/2.43*	0.77/3.17*
Kurgan	-6	-3	0		0.49/1.89*	0.83/3.42*
Omsk	n.s.	-9	0	-8		0.75/3.53*
Novosibirsk	5	-8	-6	-6	1	

### Genetic diversity of Siberian populations

**Fig 5:**
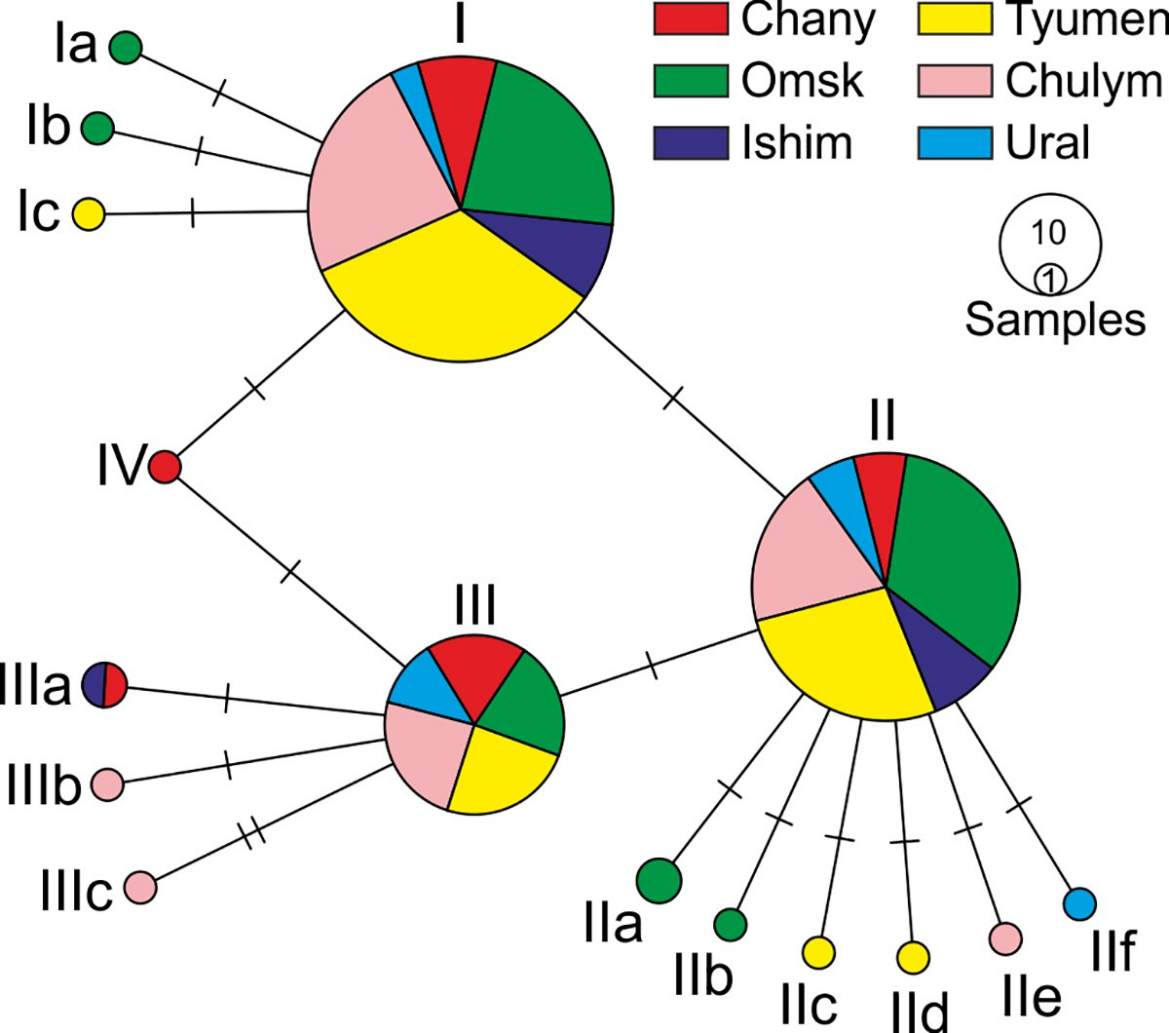
TSC network of mitochondrial haplotypes of West Siberia plain *L*. *dispar* populations. Romanian numbers mark the haplotypes numbers.

A 590-bp fragment of the *COI* gene was sequenced for 220 gypsy moth individuals collected in 18 localities of West Siberia and Trans-Ural in the 2015–2016 seasons. Genetic diversity of the studied populations was rather low. Only 14 polymorphic sites (S) were detected. Nucleotide diversity (Pi) was 0.00156 and the average genetic distance among individuals was 0.0016. Sixteen haplotypes (h) were observed with a haplotype diversity (Hd) of 0.669. Three haplotypes (I, II, and III) made up 93% of the total sample, and they were found in almost all localities (Fig 5, Table 1). Notably, these main haplotypes differed by one to two mutations (Fig 5). Haplotypes I and II were also found in other regions of the *L*. *dispar* range, including North America, Europe, and Asia [21, 22, 23, 24, 39], whereas haplotype III was unique. Negative values of Tajima’s D (-1.50781. p>0.10) and Fu’s Fs (-10.503. p = 0.0) indicated population expansion [40, 41]. The star-like network topology (Fig 5) was consistent with population expansion [42, 43]. The pairwise F_st_ values were very low, demonstrating little differentiation (Table 4). Hence, genetic data of maternal inheritance indicated that all *L*. *dispar* populations of the West Siberian plain could be considered large non-subdivided populations.

**Table 4:** Pairwise F_st_ distance between populations of *L*. *dispar*.

Population (number of samples)	Chulym	Chany	Omsk	Ishim	Tyumen
Chulym (50)	x				
Chany (21)	0.0028	x			
Omsk (58)	-0.0034	0.0357	x		
Ishim (15)	-0.0112	0.0566	-0.0155	x	
Tumen (64)	-0.0064	0.0459	0.0031	-0.0312	x
Ural (12)	0.0238	-0.0257	0.0292	0.0899	0.0779

**Fig 6:**
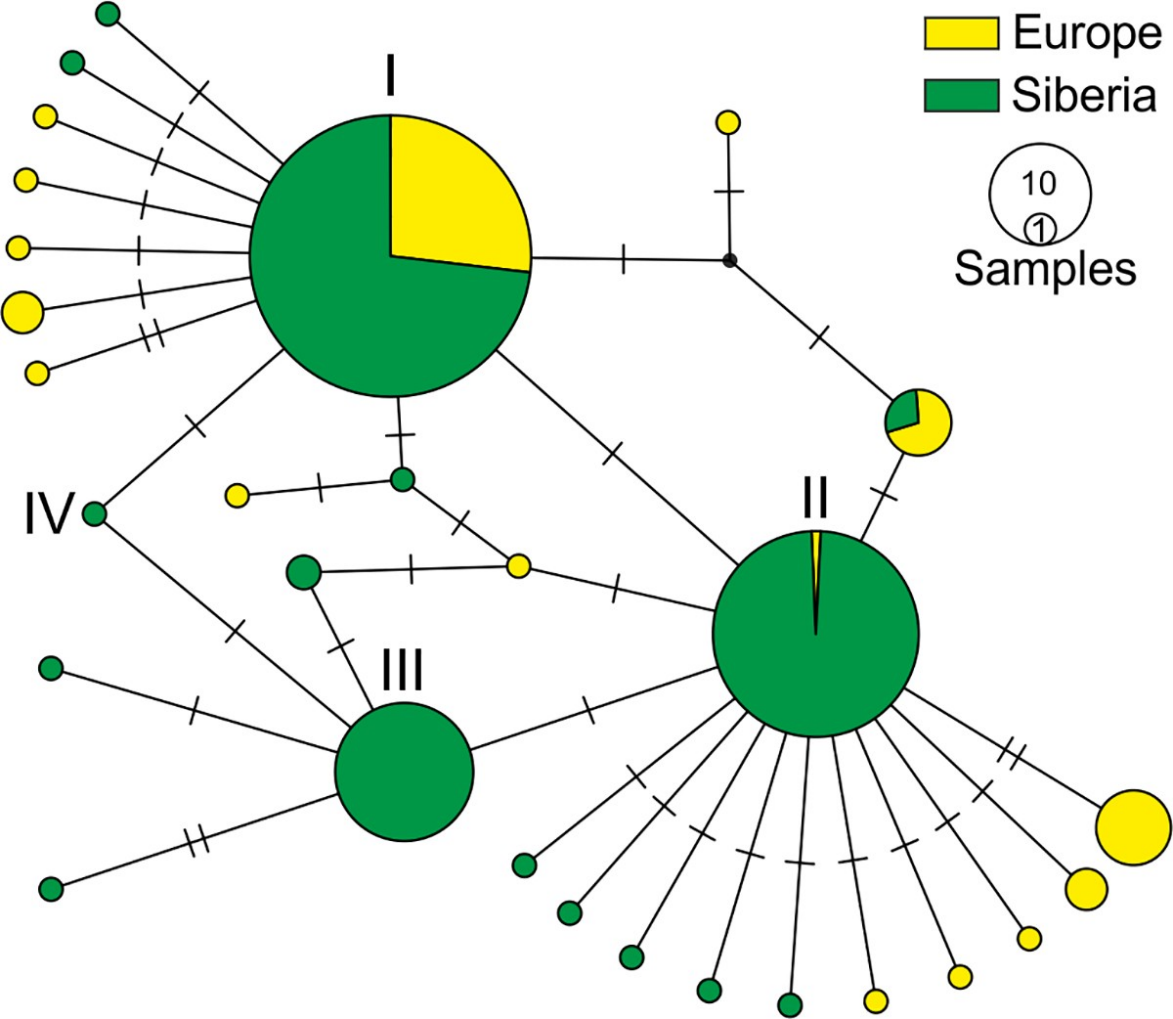
TSC network of relationships of mtDNA haplotypes of West Siberian and European *L*. *dispar* populations. Romanian numbers mark the haplotypes numbers.

To compare this phenomenon of low genetic variation of *L*. *dispar* populations in the West Siberian plain territory with other parts of the gypsy moth range, we used the data of European *L*. *dispar* populations. Although the European population sample was three-fold less than West Siberian sample, the genetic diversity of European populations was higher. In particular, S = 16, Pi = 0.00278, h = 16, Hd = 0.697, and the F_st_ between European and West Siberian populations was 0.08241. In European samples, we found that haplotype I was a major variant, haplotype II a minor variant, a few samples were the haplotype IV and there were many unique minor variants. Qualitative differences in haplotype content between these areas were exemplified by the TSC network (Fig 6). Although limitations of the data did not allow European *L*. *dispar* to be divided into different populations, the analysis indicated a noticeably larger diversity in European populations than in West Siberia.

## Discussion

Our time series results for defoliated areas clearly show that the duration of the population cycle is approximately 11 years. However for some areas population cycle was 22 years. This twice-as-long cyclicity was occurred because time series lines are restricted by only 25 variables, and the borders of *L*. *dispar* outbreaks does not well overlap with the administrative division of Russian Federation oblasts. The cross-correlation analysis readily demonstrates that most regions are characterised by the coherent trait (temporal lag in spatial context) of outbreak distributions(see Table 3). The distribution of outbreaks at a small scale (case of Novosibirsk oblast where the *L*. *dispar* outbreaks occurred in almost all administrative regions) has a directed movement in the north-east direction (Fig 4, S1 Video). Synchrony of outbreaks in one year is registered mostly for neighboring areas (S1 Table). Hence, the analysis of both large and small scales demonstrate that outbreaks taking place in West Siberia are mostly coherent, i.e., in the same period of time in different areas, we would observed wave-like distribution of *L*. *dispar* outbreaks.

The mtDNA data clearly shows that the diversity of the West Siberian populations of *L*. *dispar* is low; there are no noticeable differences among the West Siberian plain populations geographically in terms of different phases of gypsy moth population cycle. Moreover, these variants are the same (three main haplotypes, see Table 1) or closely related mtDNA haplotypes that are located in Europe and North America. Therefore, we conclude that the studied Siberian populations are not subdivided in terms of mitochondrial inheritance. A non-subdivided pattern and low mtDNA diversity for so vast a territory implies extensive genetic exchange between local populations. In contrast, the genetic diversity of European *L*. *dispar* populations is higher in comparison with West Siberian populations. This phenomenon could be explained by isolation of European populations affected by anthropogenic factors, namely pest control and others associated with the effects of urban territories in Europe. It is known that transport traffic could be also involved in the spreading of *L*. *dispar* [44, 45, 46]. However, we assume that transport is not heavily involved in the spreading of Siberian females/egg masses because: *i*) the traffic intensity and road net in Siberia is much less than in Europe while mtDNA diversity is higher in Europe; and *ii*) Egg masses laid on the transport can not successfully overwinter in Siberia. It is important to note that in Siberia egg masses of *L*. *dispar* diapause under snow layer, laying eggs near the ground (S3 Fig), i.e., eggs laid on cargos and vehicles will not be covered by snow and will not survive.

The uniform mtDNA structure of West Siberian gypsy moth populations could be explained by broad spreading of females. The best gypsy moth flyers inhabit Far East populations, where their maximum activity is estimated to be in range 1–10 km per season [18–19]. Rozkhov and Vasilyeva noted the extremely high flight abilities of L. dispar females in Siberian population [47], however, our observations over a 20-year period (Martemyanov personal observation) including several population peaks, and data published earlier [16], registered only short distance flights of female moths. We therefore suggest that, the spreading of adult females would be insufficient to explain the low diversity of such a huge region of West Siberia.

We suggest that the spread of *L*. *dispar* results mainly from the ballooning of small instar larvae on threads and this way is often used by other Lepidoptera species. For instance, such an explanation for no differences in mtDNA structure for two temporally distinct outbreaking populations of *Malacosoma californicum* was also provided by Franklin et al [48]. According to a review by Bell et al [49], the spreading distance of lepidopteran larvae by ballooning does not exceed several kilometres. In particular, the ballooning distance of European populations of *L*. *dispar* was directly estimated by net trapping and did not exceed 1 km [44, 50]. Yet, in West Siberia, hundreds of ballooned *L*. *dispar* larvae were found to be attached to electricity support poles, which were as far as 15 km from the nearest forest edge (Bakhvalov S.A. personal communication). The same scale of ballooning distance was also indirectly shown for the winter moth, *Operophtera brumata* L [51], when researchers studied the genetic structure of populations, while an earlier investigation noted much shorter ballooning distances [52].

It is significant that the spatial-temporal distribution of *L*. *dispar* outbreaks for certain regions of West Siberia occurred as a travelling wave in a north and north-east direction, as recorded over the past quarter of a century (S1 Video, Fig 4). This direction is in line with the direction of the dominant wind in this area in spring [53], when *L*. *dispar* larvae hatched [54,55]. Thus, match of wind directions and outbreak spreading and low genetic variation both indirectly reflect the importance of small larvae ballooning in open areas, like forest stands in step zone or archipelagos in open sea.

### Conclusion

We can conclude that the vast territory of West Siberia (over 1000 km from West to East) with similar climatic conditions (continental climate) and the same landscape (plain of forest-step zone) is inhabited by an non-subdivided population of *L*. *dispar* according to mtDNA data. The mtDNA diversity pattern is uniform for population cycle phases in West Siberian plain. Although the population bottle neck occurred at the trough phase, the mtDNA structure remains stable (recovers) in the following years. The following facts indicate the main role of dispersal by ballooning: *i*) the genetic similarity of mtDNA patterns of low mobility species in the vast territory; *ii*) the correspondence of the direction of outbreak movement with dominant winds; and *iii*) the observation of ballooned larvae far from a forest edge. We assume that ballooning of Lepidoptera is underestimated to date.

## Supporting information

S1 Video Visual display of directed movement of *Lymantria dispar* outbreaks in the Novosibirsk oblast. Transparent colour indicates no outbreaks in a particular district; yellow indicates that the area of the outbreak is less than 1000 ha within the district; orange—1000–10000 ha; red—more than 10000 ha.
(GIF)

S1 Fig The distribution of Lymantria dispar outbreaks in the Novosibirsk oblast, displayed as separate pictures. The numbers on the first panel indicate names of districts: 1- Kyshtovka, 2- Ust’ Tarsky, 3- Vengerovo, 4- Severny, 5- Tatarsk, 6- Chany, 7- Kuibyshev, 8- Chistoozerny, 9- Barabinsk, 10- Ubinsky, 11- Kupino, 12- Zdvinsk, 13- Kargat, 14- Bagan, 15- Karasuk, 16- Krasnozerskiy, 17- Dovol’noe, 18- Kochki, 19- Chulym, 20- Kochenevo, 21- Kolyvan’, 22- Ordynsk, 23- Novosibirsk, 24- Moshkovo, 25- Bolotnoe, 26- Suzun, 27- Iskitim, 28- Toguchin, 29- Cherepanovo 30- Maslyanino.
(PDF)

S2 Fig Numbers and localities of European collections of *Lymantria dispar* mtDNA haplotypes. (PDF)

S3 Fig Image illustrating the special feature of overwintering of Siberian populations of *Lymantria dispar*. (PDF)

S1 Table The characteristics of cross-correlation functions between comparing districts of the Novosibirsk oblast. The value of function/t-value are above the main diagonal and the temporal delay (k, years) is below.
(DOCX)

S1 Text The accession numbers of European *Lymantria dispar* specimens used for our study. (DOCX)
