# Supplementary figures and images for: Genetic evidence of broad spreading of Lymantria dispar in the West Siberian Plain
Source: PLoS One. 2019 Aug 20;14(8):e0220954. doi: 10.1371/journal.pone.0220954 (PMC6701763; doi:10.1371/journal.pone.0220954)

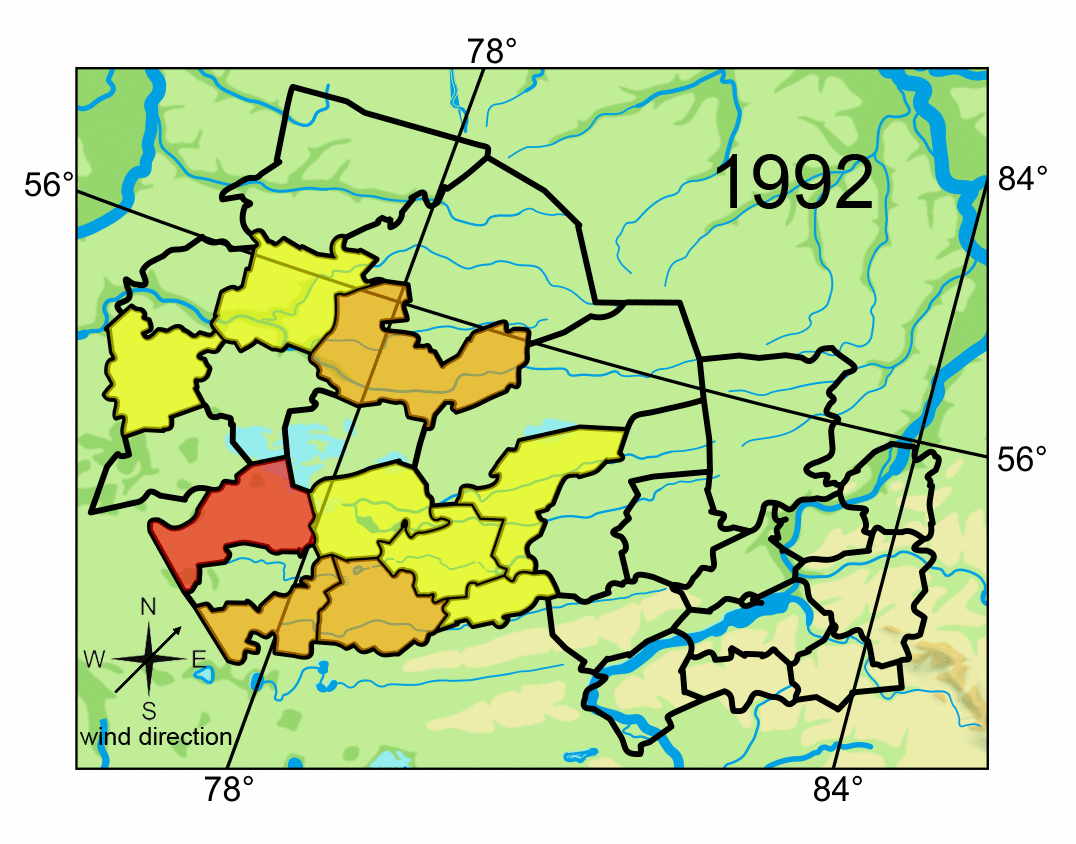

Supplement: S1 Video — Transparent colour indicates no outbreaks in a particular district; yellow indicates that the area of the outbreak is less than 1000 ha within the district; orange—1000–10000 ha; red—more than 10000 ha. (GIF) [file pone.0220954.s001.gif]

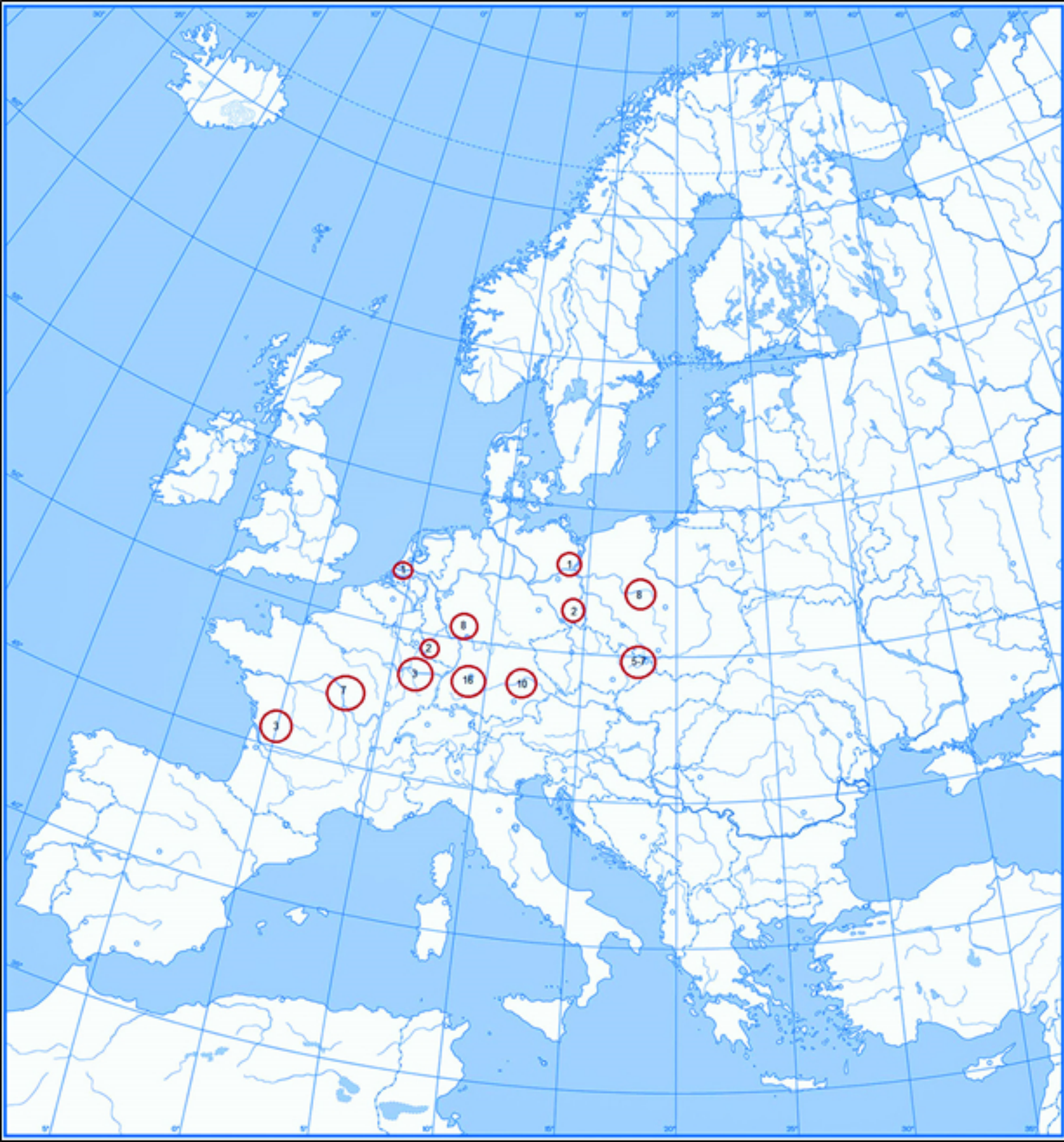

Supplement: S2 Fig — (PDF) [file pone.0220954.s003.pdf]

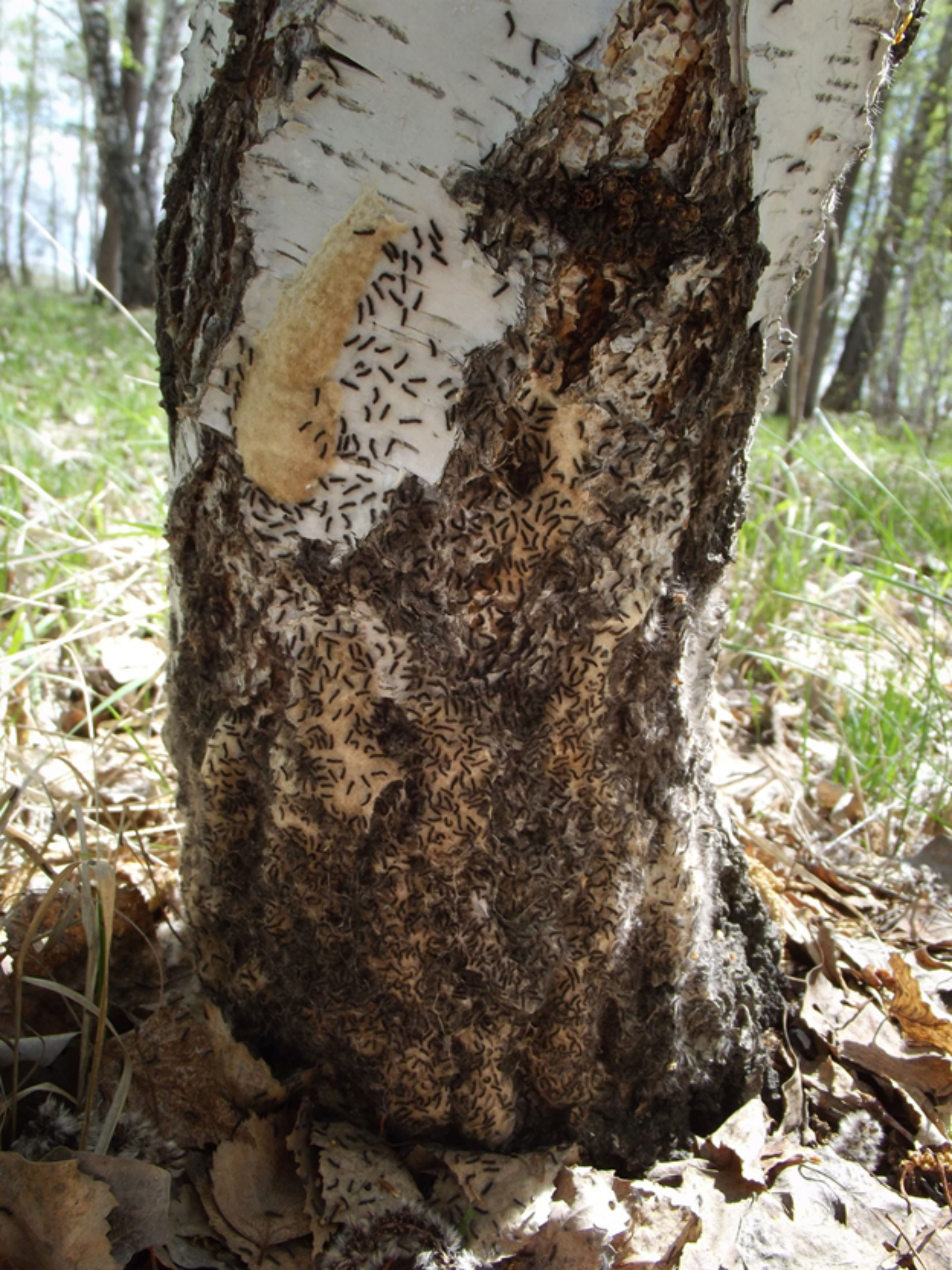

Supplement: S3 Fig — (PDF) [file pone.0220954.s004.pdf]
