# Supplementary material for: Genetic evidence of broad spreading of Lymantria dispar in the West Siberian Plain
Source: PLoS One. 2019 Aug 20;14(8):e0220954. doi: 10.1371/journal.pone.0220954 (PMC6701763; doi:10.1371/journal.pone.0220954)
Supplement: S1 Table — The value of function/t-value are above the main diagonal and the temporal delay (k, years) is below. (DOCX) [file pone.0220954.s005.docx]

|  | Vengerovo | Tatarsk | Barabinsk | Zdvinsk | Kujbyshev | Dovol’noe | Kyshtovka | Ordynskoe | Krasnozerskiy | Kupino | Karasuk |
| --- | --- | --- | --- | --- | --- | --- | --- | --- | --- | --- | --- |
| Vengerovo |  | n.s. | 0.49/2.48 | 0.53/2.57 | 0.51/2.54 | 0.62/3.12 | 0.63/3.15 | 0.74/3.75 | 0.49/2.44 | n.s. | n.s. |
| Tatarsk | n.s. |  | n.s. | n.s. | n.s. | n.s. | n.s. | n.s. | n.s. | 0.49/2.36 | 0.46/2.19 |
| Barabinsk | 0 | n.s. |  | 0.61/3.06 | 0.64/3.22 | 0.78/3.89 | 0.51/2.4 | 0.77/3.85 | 0.55/2.72 | n.s. | n.s. |
| Zdvinsk | 2 | n.s. | 0 |  | 0.65/3.16 | 0.76/3.75 | 0.68/3.28 | 0.57/2.75 | 0.71/3.48 | 0.63/3.14 | 0.58/2.92 |
| Kujbyshev | 0 | n.s. | 0 | -1 |  | 0.87/4.36 | 0.81/3.96 | 0.62/3.13 | 0.65/3.21 | 0.54/2.59 | 0.5/2.42 |
| Dovol’noe | 0 | n.s. | 0 | -1 | 0 |  | 0.71/3.41 | 0.74/3.72 | 0.69/3.39 | 0.42/2.03 | n.s. |
| Kyshtovka | 0 | n.s. | -3 | -2 | -1 | -2 |  | 0.66/3.3 | 0.55/2.63 | 0.51/2.44 | 0.44/2.06 |
| Ordynskoe | 0 | n.s. | 0 | -2 | 0 | 0 | 0 |  | 0.55/2.68 | n.s. | n.s. |
| Krasnozerskiy | 1 | n.s. | 1 | -1 | 1 | 1 | 2 | 1 |  | 0.78/3.85 | 0.74/3.65 |
| Kupino | n.s. | 2 | n.s. | 0 | 2 | 2 | 2 | n.s. | 1 |  | 0.92/4.62 |
| Karasuk | n.s. | 2 | n.s. | 0 | 2 | n.s. | 3 | n.s. | 1 | 0 |  |
