# Supplementary material for: Genetic evidence of broad spreading of Lymantria dispar in the West Siberian Plain
Source: PLoS One. 2019 Aug 20;14(8):e0220954. doi: 10.1371/journal.pone.0220954 (PMC6701763; doi:10.1371/journal.pone.0220954)
Supplement: S1 Text — (DOCX) [file pone.0220954.s006.docx]

European *Lymantria dispar* samples:

EII453-15|Lymantria dispar|COI-5P_Czech Republic Moravian-Silesian South Moravia

EII518-15|Lymantria dispar|COI-5P_Czech Republic Moravian-Silesian South Moravia

EII527-15|Lymantria dispar|COI-5P_Czech Republic Moravian-Silesian South Moravia

EII546-15|Lymantria dispar|COI-5P_Czech Republic Moravian-Silesian South Moravia

EII568-15|Lymantria dispar|COI-5P_Czech Republic Moravian-Silesian South Moravia

GBLAA1335-15|Lymantria dispar|COI-5P_Germany Bavaria Unterfranken

GBLAD102-14|Lymantria dispar|COI-5P_Germany Saxony Euba

GBLAF509-14|Lymantria dispar|COI-5P_Germany Brandenburg Barnim

GBMIN82223-17|Lymantria dispar|COI-5P|KY421535_Czech Republic

GBMIN82224-17|Lymantria dispar|COI-5P|KY421540_Czech Republic

GWOR3969-09|Lymantria dispar|COI-5P|JF415391_Germany Bavaria Oberbayern

GWORA2530-09|Lymantria dispar|COI-5P|HQ957218_Germany Bavaria Niederbayern

GWORO698-09|Lymantria dispar|COI-5P|GU688413_Germany Bavaria Oberbayern

GWOTL126-13|Lymantria dispar|COI-5P_Germany Saarland

GWOTL127-13|Lymantria dispar|COI-5P_Germany Saarland

LEFIL413-10|Lymantria dispar|COI-5P|KX041450_Germany Saxony

LYMMK043-09|Lymantria dispar|COI-5P|HM775668_France Nouvelle-Aquitaine Poitou-Charentes

LYMMK044-09|Lymantria dispar|COI-5P|HM775667_France Nouvelle-Aquitaine Poitou-Charentes

LYMMK045-09|Lymantria dispar|COI-5P|HM775666_France Nouvelle-Aquitaine Poitou-Charentes

LYMMK046-09|Lymantria dispar|COI-5P|HM775665_France Centre-Val de Loire Centre

LYMMK048-09|Lymantria dispar|COI-5P|HM775664_France Centre-Val de Loire Centre

LYMMK049-09|Lymantria dispar|COI-5P|HM775663_France Centre-Val de Loire Centre

LYMMK050-09|Lymantria dispar|COI-5P|HM775662_France Centre-Val de Loire Centre

LYMMK051-09|Lymantria dispar|COI-5P|HM775661_France Centre-Val de Loire Centre

LYMMK052-09|Lymantria dispar|COI-5P|HM775660_France Centre-Val de Loire Centre

LYMMK053-09|Lymantria dispar|COI-5P|HM775659_France Centre-Val de Loire Centre

LYMMK054-09|Lymantria dispar|COI-5P|HM775658_France Grand Est Alsace

LYMMK055-09|Lymantria dispar|COI-5P|HM775657_France Grand Est Alsace

LYMMK056-09|Lymantria dispar|COI-5P|HM775656_France Grand Est Alsace

LYMMK057-09|Lymantria dispar|COI-5P|HM775655_Germany Baden-Wuerttemberg

LYMMK058-09|Lymantria dispar|COI-5P|HM775654_Germany Baden-Wuerttemberg

LYMMK059-09|Lymantria dispar|COI-5P|HM775653_Germany Baden-Wuerttemberg

LYMMK060-09|Lymantria dispar|COI-5P|HM775652_Germany Baden-Wuerttemberg

LYMMK061-09|Lymantria dispar|COI-5P|HM775651_Germany Baden-Wuerttemberg

LYMMK062-09|Lymantria dispar|COI-5P|HM775650_Germany Baden-Wuerttemberg

LYMMK063-09|Lymantria dispar|COI-5P|HM775649_Germany Baden-Wuerttemberg

LYMMK064-09|Lymantria dispar|COI-5P|HM775648_Germany Baden-Wuerttemberg

LYMMK065-09|Lymantria dispar|COI-5P|HM775647_Germany Baden-Wuerttemberg

LYMMK066-09|Lymantria dispar|COI-5P|HM775646_Germany Baden-Wuerttemberg

LYMMK067-09|Lymantria dispar|COI-5P|HM775645_Germany Baden-Wuerttemberg

LYMMK068-09|Lymantria dispar|COI-5P|HM775644_Germany Baden-Wuerttemberg

LYMMK069-09|Lymantria dispar|COI-5P|HM775643_Germany Baden-Wuerttemberg

LYMMK070-09|Lymantria dispar|COI-5P|HM775642_Germany Baden-Wuerttemberg

LYMMK071-09|Lymantria dispar|COI-5P|HM775641_Germany Baden-Wuerttemberg

LYMMK072-09|Lymantria dispar|COI-5P|HM775640_Germany Baden-Wuerttemberg

LYMMK073-09|Lymantria dispar|COI-5P|HM775606_Germany Bavaria

LYMMK074-09|Lymantria dispar|COI-5P|HM775639_Germany Bavaria

LYMMK075-09|Lymantria dispar|COI-5P|HM775638_Germany Bavaria

LYMMK076-09|Lymantria dispar|COI-5P|HM775637_Germany Hesse

LYMMK078-09|Lymantria dispar|COI-5P|HM775636_Germany Hesse

LYMMK079-09|Lymantria dispar|COI-5P|HM775635_Germany Bavaria

LYMMK080-09|Lymantria dispar|COI-5P|HM775634_Germany Bavaria

LYMMK081-09|Lymantria dispar|COI-5P|HM775633_Germany Bavaria

LYMMK082-09|Lymantria dispar|COI-5P|HM775632_Germany Hesse

LYMMK083-09|Lymantria dispar|COI-5P|HM775631_Germany Hesse

LYMMK084-09|Lymantria dispar|COI-5P|HM775630_Germany Hesse

LYMMK085-09|Lymantria dispar|COI-5P|HM775629_Germany Hesse

LYMMK086-09|Lymantria dispar|COI-5P|HM775628_Germany Hesse

LYMMK087-09|Lymantria dispar|COI-5P|HM775627_Germany Hesse

LYMMK100-09|Lymantria dispar|COI-5P|HM775600_Poland Poznan Region

LYMMK102-09|Lymantria dispar|COI-5P|HM775599_Poland Poznan Region

LYMMK103-09|Lymantria dispar|COI-5P|HM775598_Poland Poznan Region

LYMMK104-09|Lymantria dispar|COI-5P|HM775597_Poland Poznan Region

LYMMK105-09|Lymantria dispar|COI-5P|HM775596_Poland Poznan Region

LYMMK106-09|Lymantria dispar|COI-5P|HM775610_Poland Poznan Region

LYMMK107-09|Lymantria dispar|COI-5P|HM775609_Poland Poznan Region

LYMMK108-09|Lymantria dispar|COI-5P|HM775595_Poland Poznan Region

NLLEA1111-12|Lymantria dispar|COI-5P|KX049315_Netherlands South Holland
