# Supplementary material for: Genetic evidence of broad spreading of Lymantria dispar in the West Siberian Plain
Source: PLoS One. 2019 Aug 20;14(8):e0220954. doi: 10.1371/journal.pone.0220954 (PMC6701763; doi:10.1371/journal.pone.0220954)
Supplement: S1 Fig — The numbers on the first panel indicate names of districts: 1- Kyshtovka, 2- Ust’ Tarsky, 3- Vengerovo, 4- Severny, 5- Tatarsk, 6- Chany, 7- Kuibyshev, 8- Chistoozerny, 9- Barabinsk, 10- Ubinsky, 11- Kupino, 12- Zdvinsk, 13- Kargat, 14- Bagan, 15- Karasuk, 16- Krasnozerskiy, 17- Dovol’noe, 18- Kochki, 19- Chulym, 20- Kochenevo, 21- Kolyvan’, 22- Ordynsk, 23- Novosibirsk, 24- Moshkovo, 25- Bolotnoe, 26- Suzun, 27- Iskitim, 28- Toguchin, 29- Cherepanovo 30- Maslyanino. (PDF) [file pone.0220954.s002.pdf]

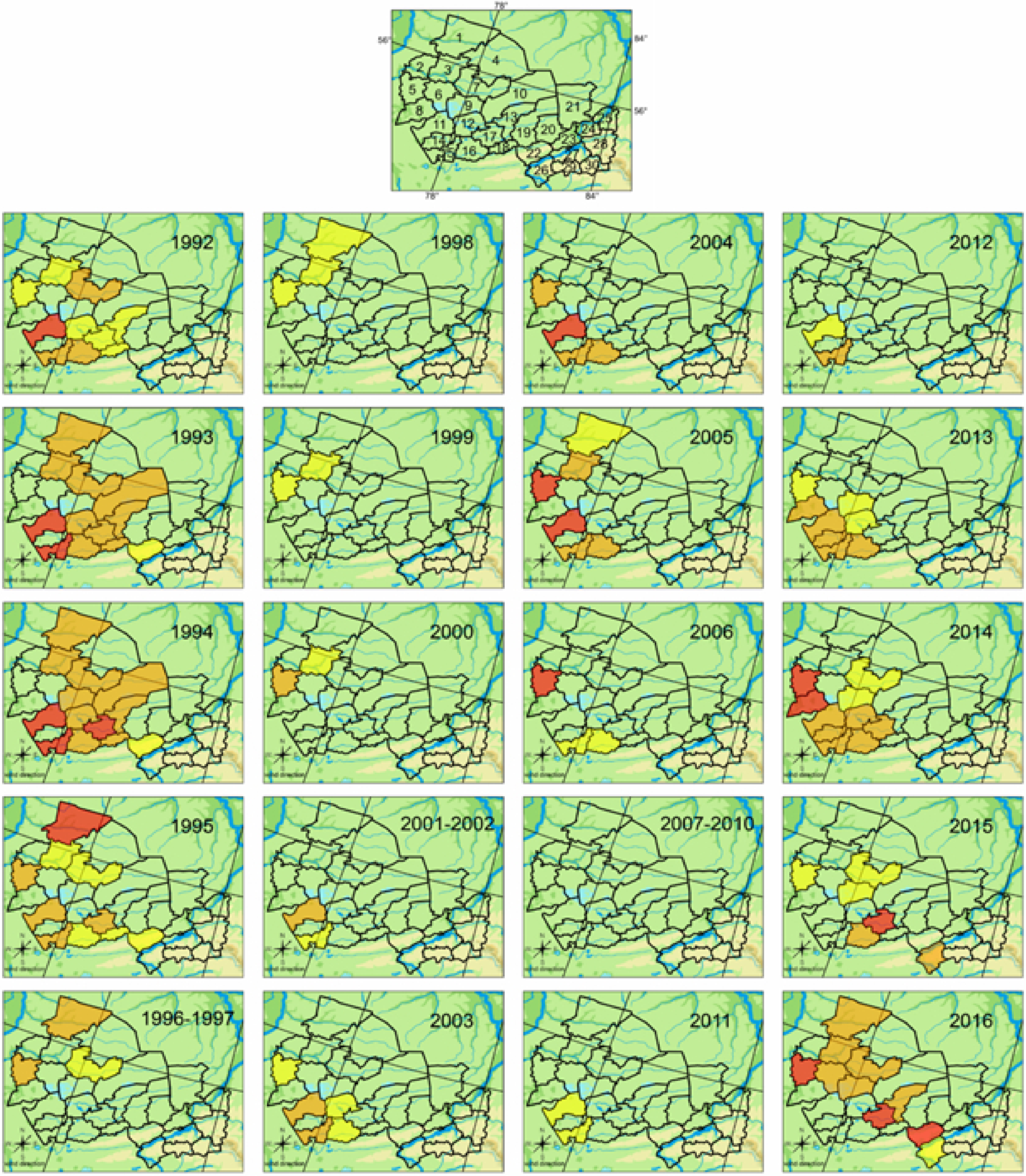

78°
56°
84°
56°
78°
84°
1
2
3
4
5
6
7
8
9
10
11
12
13
14
15
16
17
19
20
21
22
23
24
25
26
28
1992
1998
2004
2012
1993
1999
2005
2013
1994
2000
2006
2014
1995
2001-2002
2007-2010
2015
1996-1997
2003
2011
2016
